# Novel gene rearrangement in the mitochondrial genome of *Muraenesox cinereus* and the phylogenetic relationship of Anguilliformes

**DOI:** 10.1038/s41598-021-81622-9

**Published:** 2021-01-28

**Authors:** Kun Zhang, Kehua Zhu, Yifan Liu, Hua Zhang, Li Gong, Lihua Jiang, Liqin Liu, Zhenming Lü, Bingjian Liu

**Affiliations:** 1 National Engineering Laboratory of Marine Germplasm Resources Exploration and Utilization, Zhejiang Ocean University, No. 1, Haida South Road, Zhoushan, Zhejiang 316022 People’s Republic of China; 2 Key Laboratory of Tropical Marine Bio-Resources and Ecology, Chinese Academy of Sciences, Beijing, People’s Republic of China; 3 National Engineering Research Center for Facilitated Marine Aquaculture, Marine Science and Technology College, Zhejiang Ocean University, Zhoushan, 316022 People’s Republic of China

**Keywords:** Evolutionary genetics, Molecular evolution, Evolutionary biology, Genomics, Evolution, Genetics, Molecular biology, DNA recombination, Transposition, Ecology, Biodiversity, Ecological genetics, Evolutionary ecology, Molecular ecology

## Abstract

The structure and gene sequence of the fish mitochondrial genome are generally considered to be conservative. However, two types of gene arrangements are found in the mitochondrial genome of Anguilliformes. In this paper, we report a complete mitogenome of *Muraenesox cinereus* (Anguilliformes: Muraenesocidae) with rearrangement phenomenon. The total length of the *M. cinereus* mitogenome was 17,673 bp, and it contained 13 protein-coding genes, two ribosomal RNAs, 22 transfer RNA genes, and two identical control regions (CRs). The mitochondrial genome of *M. cinereus* was obviously rearranged compared with the mitochondria of typical vertebrates. The genes *ND6* and the conjoint *trnE* were translocated to the location between *trnT* and *trnP*, and one of the duplicated CR was translocated to the upstream of the *ND6*. The tandem duplication and random loss is most suitable for explaining this mitochondrial gene rearrangement. The Anguilliformes phylogenetic tree constructed based on the whole mitochondrial genome well supports Congridae non-monophyly. These results provide a basis for the future Anguilliformes mitochondrial gene arrangement characteristics and further phylogenetic research.

## Introduction

Anguilliformes is a kind of ecologically diverse fish, mainly marine fish. Its body is very slender, its cross-sectional area is reduced, and it generally lacks ventral fins^1,2^. Traditional morphological taxonomy divides the eel-shaped order (the largest one) into three sub-orders: Anguilloidei, Congroidei and Muraenoidei^3^. However, previous phylogenetic studies of Anguilliformes relationship based solely on morphological data have failed to resolve the relationship between these three suborders^3–5^. For example, recent molecular analysis based on mitochondrial and nucleic acid data has raised questions about the taxonomic status of Anguilliformes^6,7^. The phylogenetic relationship of Anguilliformes is still unclear. In particular, the family-level classification needs to be revised, because many known families or genera may be multi-lineage, especially the well-known Congridae and Nettastomatidae^8,9^. *M. cinereus* belongs to the *Muranesox* genus in the Muranesocidae family, also known as dagger-tooth pike conger. *M. cinereus* is located as far north as Japan and South Korea, and south as far as the Arafara Sea and North Australia^10^. *M. cinereus* mainly inhabits soft bottoms, but was also common in estuaries. *M. cinereus* was an important economic fish and one of the most popular moray eels in China^11^.

Mitochondria was the two layered organelles found in most cells. It was also producing energy in the cell structure and the major site of aerobic respiration. So it was called “power house”. The length of vertebrate mitochondria was 16–20 kb, which was usually a typical circular structure, containing 13 protein coding genes (PCGs), 22 tRNA genes (tRNA) and two rRNA genes (rRNA)^12,13^. The order of these genes was initially considered conservative because the mammalian complete mitochondrial genome and the African clawed frog have the same genetic order^14–16^. Mitochondrial genomes were maternally inherited, so the recombination rate was extremely low, and the replacement rate was faster than that of nuclear DNA^17^. Therefore, mitochondrial markers have become the most commonly used inference target for molecular phylogeny of fish species. However, variations in this gene sequence have been found in various vertebrate lineages, including amphibians^18,19^, reptiles^20,21^, birds^22,23^, marsupials^24^ and fish^8,13,25^. In recent years, with the increase in the number of mitochondrial genome sequencing and the improvement of technology, people have discovered more and more gene rearrangements^19–21^. However, the bony fish with the largest number of published complete mitochondrial genomes showed only a partial gene rearrangement^26–28^. Gong^29^ summarized the complete sequence of 1255 fish mitochondrial genomes in the National Center for Biotechnology Information (NCBI) database, and found that 52 fish species have rearranged mitochondrial genomes, including shuffling, translocation, and inversion, involving 15 subjects and 34 families. The probability of rearrangement of genes in the fish mitochondrial genome is low.

In general, the mitochondrial genome structure of fish, especially in the order of genes, is highly conserved^29^. However, with the gradual increase of mtDNA sequence data of fish, there have been reports of rearrangement of mitogenome^18,30–32^. In general, a bony fish group has only one or a group of similar mitochondrial gene arrangements^33^. However, after comparing the gene rearrangement of the mitochondria of Anguilliformes, we found that there are two different gene rearrangement phenomena: (1) most have the typical vertebrate gene sequence (2) in a few mitochondrial genomes, the *ND6* gene combines with *trnE* was transferred to the position between *trnT* and *trnP*, and accompanied by repetition of CR^13^.

So far, four main hypotheses have been proposed to explain the gene rearrangement in animal mitochondrial genomes. The first hypothesis is that Poulton first proposed a model of in-patient mitochondrial reorganization when studying patient mitochondria, which is characterized by involvement in DNA strand breakage and reconnection^34^; this hypothesis was originally proposed for gene rearrangement in the nuclear genome^35^. This gene rearrangement model has been used to explain changes in the mitochondrial gene order of mussels, birds and frogs^36–38^. Another commonly accepted assumption is the tandem replication and random loss (TDRL) model, which assumes that rearrangement occurs through the tandem replication of some genes, followed by random deletion of duplicates^39,40^. This model is used to explain the gene rearrangement of vertebrate mitochondrial genome^41,42^. Lavrov et al^43^. proposed this rearrangement model (the tandem duplication and non-random loss) for the first time to explain the mitochondrial gene rearrangement of two kinds of millipedes. The difference between this model and the TDRL model is that this loss is non-random, and it depends on the transcription polarity and location of the gene. Shi et al^27^. raised a new model, the double replication random loss (DRRL) model to explain the rearrangement of the flatfish *Samariscus latus* (Samaridae) genome. According to this model, the control region (CR) was usually copied and shifted. Then, the two CRs successively initiated the double replication of the mitochondrial genome, leading to gene replication between the two CRs. Finally, one of each pair of duplicated genes was randomly lost^27^.

Previous studies have shown that rearrangement of the mitochondrial genome can provide important clues to the evolution and origin of species^44,45^. In this paper, the gene structure and gene rearrangement of the mitochondrial genome of *M. cinereus* (Anguilliformes, Muraenesocidae) common in Chinese waters were reported, and the relationship between the mitochondrial genome rearrangement and phyletic evolution of Anguilla was further discussed based on previous report. Based on the similarities and differences of the gene rearrangement order in the mitochondrial genome, the possible rearrangement process was discussed in order to have a better understanding of the rearrangement events and evolutionary mechanisms of the eel mitochondrial genome.

## Results and discussion

### Genome structure and composition

**Figure 1:**
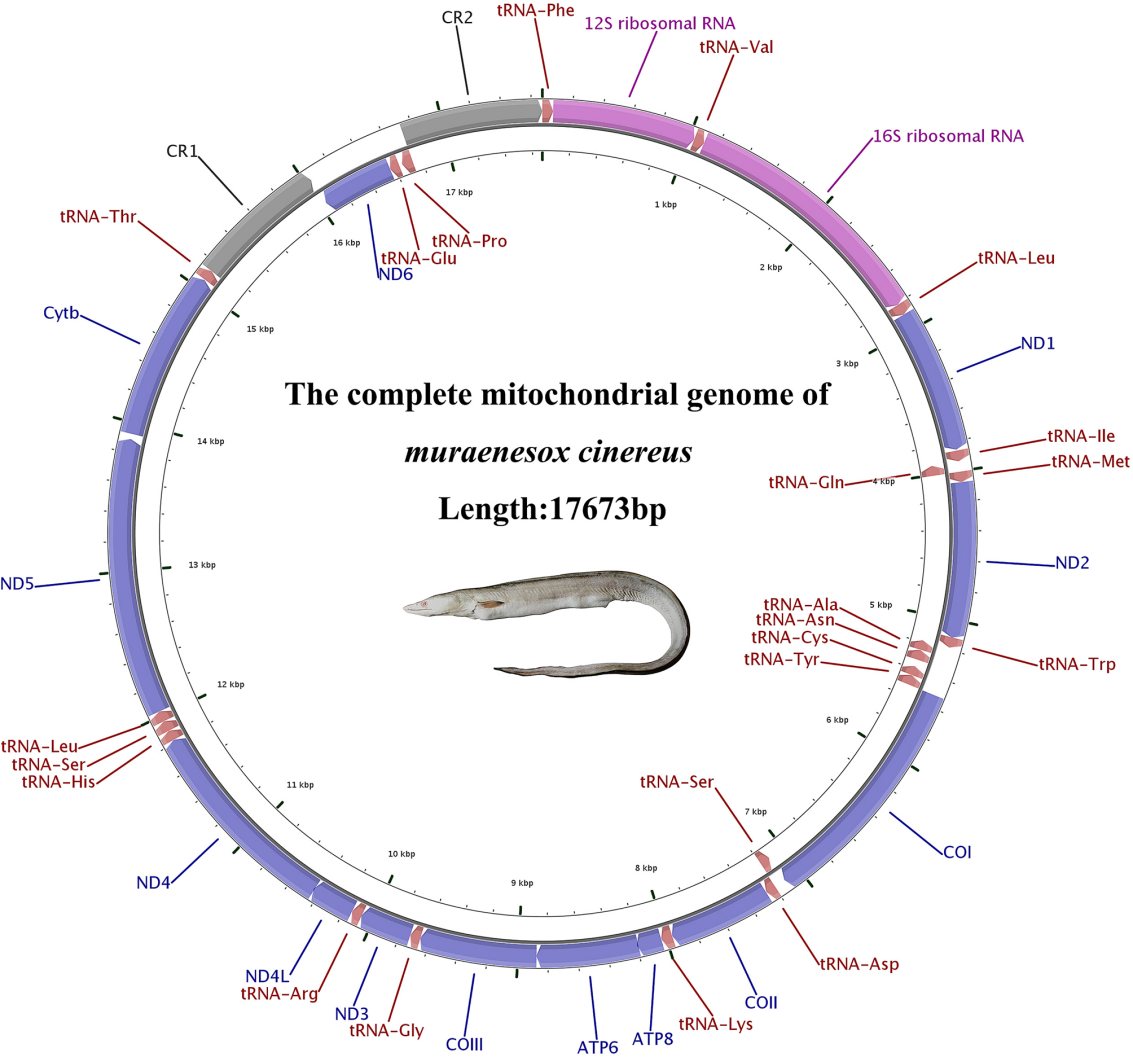
*Muraenesox cinereus* mitochondrial genome visualization ring diagram.

The complete mitochondrial genome of *M. cinereus* is 17,673 bp (GenBank accession number MT571331), which is within the published length range of the bony fish’s mitochondrial genome (16,417–18,369 bp) (Table 1). The structure of the moray mitochondrial genome was different from other bony fishes, it includes 13 PCGs, 22 tRNAs, two rRNAs (12S and 16S rRNA), a light chain replication source (O_L_) and two control-region s (CR) (Fig. 1). And the gene rearrangement was different from some other fish mitochondria. To be precise, *ND6* binding *trnE* was transferred between *tRNAT* and *tRNAP*, and a replicated CR was transferred upstream of the *ND6* gene (Fig. 1, Table 2). In the vertebrate mitochondrial genome, the presence of replicated CR was considered a special feature^46–48^. The base composition of *M. inereus* mitochondrial genome was: A = 32.1%, T = 27.6%, C = 23.8% and G = 16.6%, respectively (Table 3). Overall, the mitotic genome was very compact. However, 89 base pairs of 12 gene spacers were found in the mitochondrial genome of moray eel, ranging in length from 1 to 35 bp. Most of the gaps were found in the area where rearrangement occurred, including 35 bp between *Cytb* and *trnT*, 4 bp between *trnT* and *CR1*, 1 bp between *trnE* and *trnP*, and 23 bp between CR2 and *trnP*. The AT-skew of the mitochondrial genome was positive and the GC-skew was negative, respectively 0.076 and -0.179, indicating that As and Cs are more abundant than Ts and Gs.

**Table 1:** List of 44 Anguilliformes species and 2 outgroups used in this paper.

Family	Species	Length (bp)	Accession No	References
Nemichthyidae	*Nemichthys scolopaceus*	17,457	NC_013620	^64^
	*Labichthys carinatus*	16,683	NC_013626	^64^
Serrivomeridae	*Serrivomer sector*	16,099	NC_013436	^13^
	*Stermonidium hypomelas*	16,566	NC_013628	^13^
Anguillidae	*Anguilla dieffenbachi*	16,687	NC_06538	^13^
	*Anguilla megastoma*	16,714	NC_006541	^13^
	*Anguilla japonica*	16,685	NC_002707	^13^
	*Anguilla reinhardti*	16,690	NC_006546	^13^
	*Anguilla marmorata*	16,745	NC_06540	^13^
	*Anguilla interioris*	16,713	NC_006539	^13^
	*Anguilla obscura*	16,704	NC_006545	^13^
	*Anguilla bicolor bicolor*	16,700	NC_006534	^13^
	*Anguilla bicolor pacifica*	16,693	NC_065035	^13^
Moringuidae	*Moringua microchir*	15,858	NC_013602	^13^
	*Moringua edwardsi*	16,841	NC_013622	^64^
Chlopsidae	*Kaupichthys hyoproroides*	16,662	NC_013607	^64^
	*Robinsia catherinae*	16,627	NC_013633	^64^
Synaphobranchidae	*Simenchelys parasitica*	16,689	NC_013605	^64^
	*Synaphobranchus kaupli*	16,166	NC_005805	^13^
	*Ilyophis brunneus*	16,682	NC_013634	^13^
Heterenchelyidae	*Pyhonichthys microphthalmus*	17,042	NC_013601	^13^
Myrocongridae	*Myroconger compressus*	16,642	NC_013631	^64^
Muraenidae	*Scuticaria tigrina*	16,521	KP874183	^13^
	*Gymnomuroena zebra*	16,576	NC_027240	^13^
	*Gymnothorax formosus*	16,558	KP874184	^13^
	*Gymnnothorax kidako*	16,579	NC_04417	^13^
	*Rhinomuroena quaesita*	16,566	NC_013610	^13^
	*Gymnothorax minor*	16,574	NC_038175	Unpublished
Derichthyidae	*Derichthys serpentinus*	17,077	NC_013611	^64^
	*Coloconger cadenati*	17,755	NC_013606	^64^
	*Nessorhamphus inglfianus*	17,782	NC_013608	^64^
Nettastomatidae	*Facciolella oxyrhyncha*	17,789	NC_013621	^64^
	*Hoplunnis punctota*	17,828	NC_013623	^64^
	*Leptocephalus sp*	18,037	NC_013615	^64^
	*Nettastoma parviceps*	17,714	NC_013625	^64^
Congridae	*Conger myriaster*	18,705	NC_002761	^8^
	*Conger japonicu*	17,778	NC_027186	^64^
	*Heteroconger hassi*	17,768	NC_013629	^64^
	*Paraconger notialis*	17,729	NC_013630	^64^
Muraenesocidae	*Muraenesox bogio*	18,247	NC_013614	^64^
	*Muraenesox cinereus*	17,673	MT571331	This study
Ophichthidae	*Ophichthus rotundus*	17,785	KY081397	^64^
	*Ophichthus brevirostris*	17,773	MK189459	^13^
	*Ophisurus macrorhynchos*	17,843	NC_005802	^13^
	*Myrichthys maculosus*	17,859	NC_013635	^64^
Eurypharyngidae	Eurypharynx pelecanoides	18,978	NC_005299	^63^
Saccopharyngidae	Saccopharynx lavenbergi	18,495	NC_005298	^63^

**Table 2:** Features of the mitochondrial genome of *Muraenesox cinereus.*

Gene	Position		Length (bp)	Amino acid	Start/Stop codon	Anticodon	Intergenic region (bp)	Strand
	From	To						
*tRNA-Phe* (*F*)	1	72	72			GAA	0	H
*12S RNA*	73	1034	962				0	H
*tRNA-Val* (*V*)	1035	1104	70			TAC	0	H
*16S RNA*	1105	2810	1706				0	H
*tRNA-Leu*^UUA^ (*L*_*1*_)	2811	2886	76			TAA	0	H
*ND1*	2887	3855	969	323	ATG/TAA		0	H
*tRNA-Ile* (*I*)	3863	3934	72			GAT	7	H
*tRNA-Gln* (*Q*)	3935	4005	71				0	L
*tRNA-Met* (*M*)	4005	4074	70			CAT	− 1	H
*ND2*	4075	5118	1044	348	ATG/TAG		0	H
*tRNA-Trp* (*W*)	5117	5186	70			TCA	-2	H
*tRNA-Ala* (*A*)	5188	5256	69			TGC	1	L
*tRNA-Asn* (*N*)	5258	5330	73			GTT	1	L
O_L_	5331	5369	39				− 7	H
*tRNA-Cys* (*C*)	5381	5446	66			GCA	7	L
*tRNA-Tyr* (*Y*)	5447	5517	71			GCA	0	L
*COI*	5519	7121	1603	534	GTG/TAA		1	H
*tRNA-Ser*^UCA^ (*S*_*1*_)	7122	7192	71			TGA	0	L
*tRNA-Asp* (*D*)	7198	7265	68			GTC	5	H
*COII*	7269	7959	691	230	ATG/T		3	H
*tRNA-Lys* (*K*)	7960	8034	75			TTT	0	H
*ATP8*	8036	8203	168	56	ATG/TAA		1	H
*ATP6*	8194	8877	684	228	ATG/TAA		− 10	H
*COIII*	8877	9662	786	262	ATG/TAA		− 1	H
*tRNA-Gly* (*G*)	9662	9731	70			TCC	− 1	H
*ND3*	9732	10,082	351	117	ATG/TAG		0	H
*tRNA-Arg* (*R*)	10,081	10,150	70			TCG	− 2	H
*ND4L*	10,151	10,447	297	99	ATG/TAA		0	H
*ND4*	10,441	11,821	1381	460	ATG/T		− 7	H
*tRNA-His* (*H*)	11,822	11,890	69			GTG	0	H
*tRNA-Ser*^AGC^ (*S*_*2*_)	11,891	11,961	71			GCT	0	H
*tRNA-Leu*^CUA^ (*L*_*2*_)	11,962	12,033	72			TAG	0	H
*ND5*	12,034	13,890	1857	619	ATG/TAA		0	H
*Cyt b*	13,926	15,065	1140	380	ATG/AGA		35	H
*tRNA-Thr* (*T*)	15,070	15,141	72			TGT	4	H
CR1	15,142	16,043	902				0	H
*ND6*	16,044	16,559	516	172	ATG/AGG		0	L
*tRNA-Glu* (*E*)	16,561	16,629	69			TTC	1	L
*tRNA-Pro* (*P*)	16,653	16,729	77			TGG	23	L
CR2	16,730	17,673	944				0	H

**Table 3:** Composition and skewness of *Muraenesox cinereus* mitogenome.

	A	T	C	G	A + T%	AT-skew	GC-skew	Length (bp)
Mitogenome	32.1	27.6	23.8	16.6	59.7	0.076	− 0.179	17,673
*ND1*	28.8	28.6	26.9	15.7	57.4	0.004	− 0.264	969
*ND2*	34.9	28.7	24.0	12.4	63.6	0.096	− 0.321	1044
*COI*	26.4	31.1	24.0	18.5	57.5	− 0.082	-0.128	1603
*COII*	30.1	28.5	24.5	16.9	58.6	0.027	− 0.182	691
*ATP8*	35.1	30.4	25.6	8.9	65.5	0.073	− 0.483	168
*ATP6*	30.3	32.6	24.4	12.7	62.9	− 0.037	− 0.315	684
*COIII*	28.1	30.7	23.9	17.3	58.8	− 0.043	− 0.160	786
*ND3*	28.2	33.3	24.8	13.7	61.5	− 0.083	− 0.289	351
*ND4*	31.3	29.8	23.6	15.4	61.0	0.025	− 0.212	1381
*ND4L*	25.9	27.6	29.3	17.2	53.5	− 0.031	− 0.261	297
*ND5*	32.5	28.4	25.5	13.6	60.9	0.067	− 0.304	1857
*Cytb*	28.7	27.9	26.4	17.0	56.6	0.014	− 0.216	1140
*ND6*	44.6	14.1	28.3	13	58.7	0.520	− 0.370	516
*tRNA*	32.0	25.3	23.1	19.6	57.3	0.116	− 0.081	1564
*rRNA*	35.1	21.7	22.6	20.6	56.7	0.236	− 0.047	2668
CR	36.2	30.1	18.0	15.8	66.3	0.092	− 0.066	1846

### PCGs and codon usage

The total length of the 13 PCGs in the *M. cinereus* mitochondrial genes were 11,454 bp, and they encode 3,818 amino acids. Genes encoding 13 proteins include seven NADH dehydrogenases (*ND1-ND6* and *ND4L*), three cytochrome c oxidases (*COI-COII*), two ATPases (*ATP6* and *ATP8*) and one cytochrome b (*cytb*). The 13 PCGs range in size from 168 bp (*ATP8*) to 1857 bp (*ND5*) (Table 3). Like the typical mitochondrial genome of vertebrates^49,50^, there are twelve genes in the H-strand and only *ND6* genes in the L-strand.

The initiation codon of the 13 protein-coding genes used the typical initiation codon ATG, except for GTG for the *COI* gene. Seven PCGs (*ND1*, *COI*, *ATP8*, *ATP6*, *COIII*, *ND4L*, and *ND5*) were terminated with a stop codon TAA, three (*ND2*, *ND3*, and *ND6*) were terminated with TAG, and one (*Cyt b*) was terminated with AGA, in addition, *ND4* And *COII* terminate with an incomplete nucleotide T- (Table 2). This result was very similar to that of Lu et al^13^. Whether in invertebrate or vertebrate mitochondrial genes, the presence of incomplete stop codons is a common phenomenon^51–53^. For genes with TAA as the stop codon, one of the most common interpretations is that produced by polyadenylation after transcription^54^. In the 13 PCGS, the values of *COI*, *ATP6*, *COII*, *ND3*, and *ND4* of the AT-skew and GC-skew are negative, and the rest is both positive and GC-skew is negative (Table 3).

**Figure 2:**
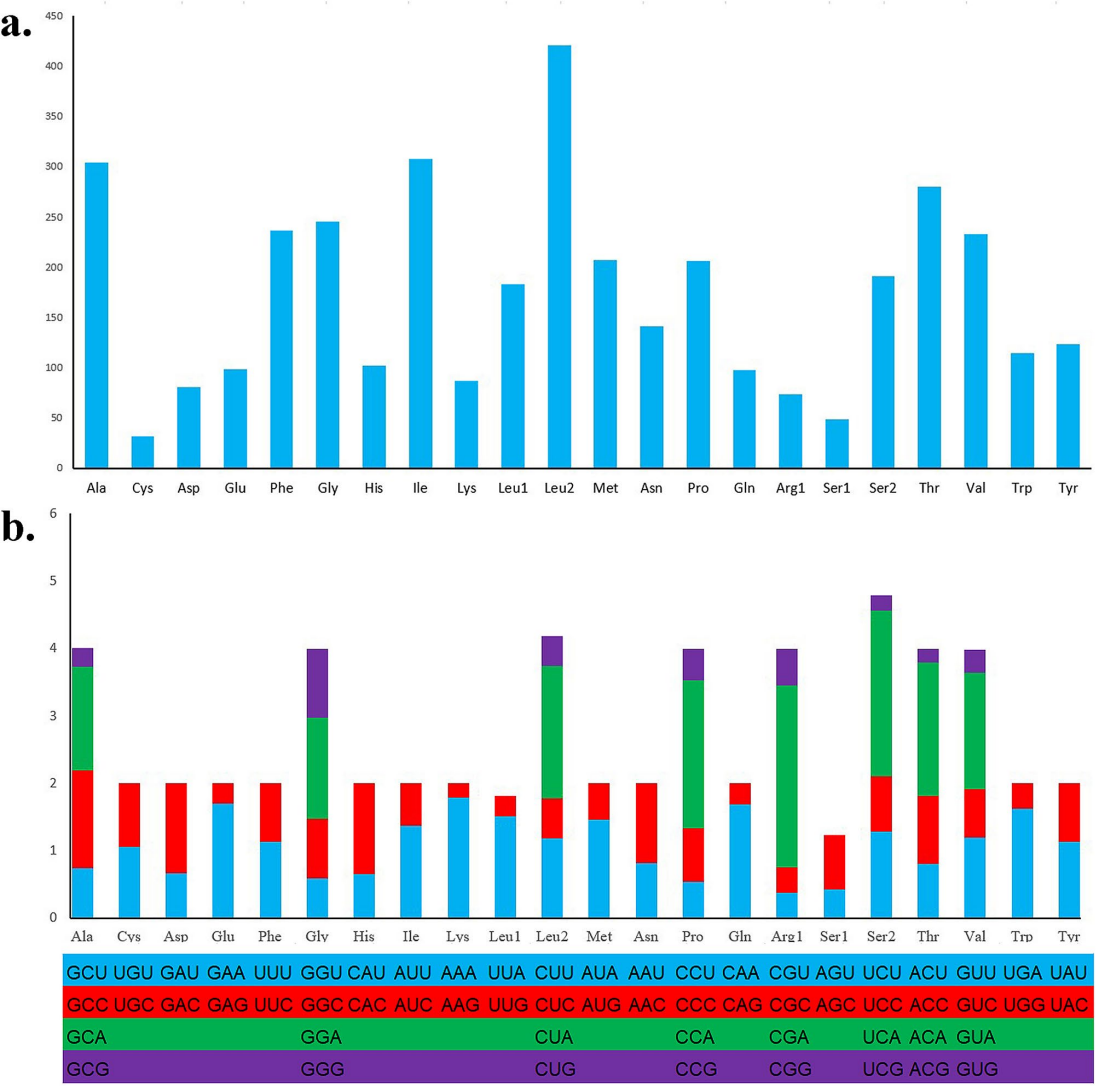
Amino acid composition (**a**) and Relative synonymous codon usage (**b**) in *Muraenesox cinereus* mitogenome.

According to the codon degeneracy pattern, the amino acids serine and leucine are encoded by six synonymous codons, and the remaining amino acids are encoded by four or two codons. This result also appears in the research results of Vandana et al^55^. The most used amino acids are Leu (15.82%), Ile (8.07%) and Ala (7.96%), the few most used amino acids are Asp (2.12%), Arg (1.94%) and Cys (0.84%) (Fig. 2a). The relative synonymous codon usage (RSCU) value for *M. cinereus* for the third position is shown in Fig. 2b. The usage of both two- and four-fold degenerate codons was biased toward the use of codons abundant in A, while there was an overall bias against G.

### Transfer RNAs, ribosomal RNAs and CR

**Figure 3:**
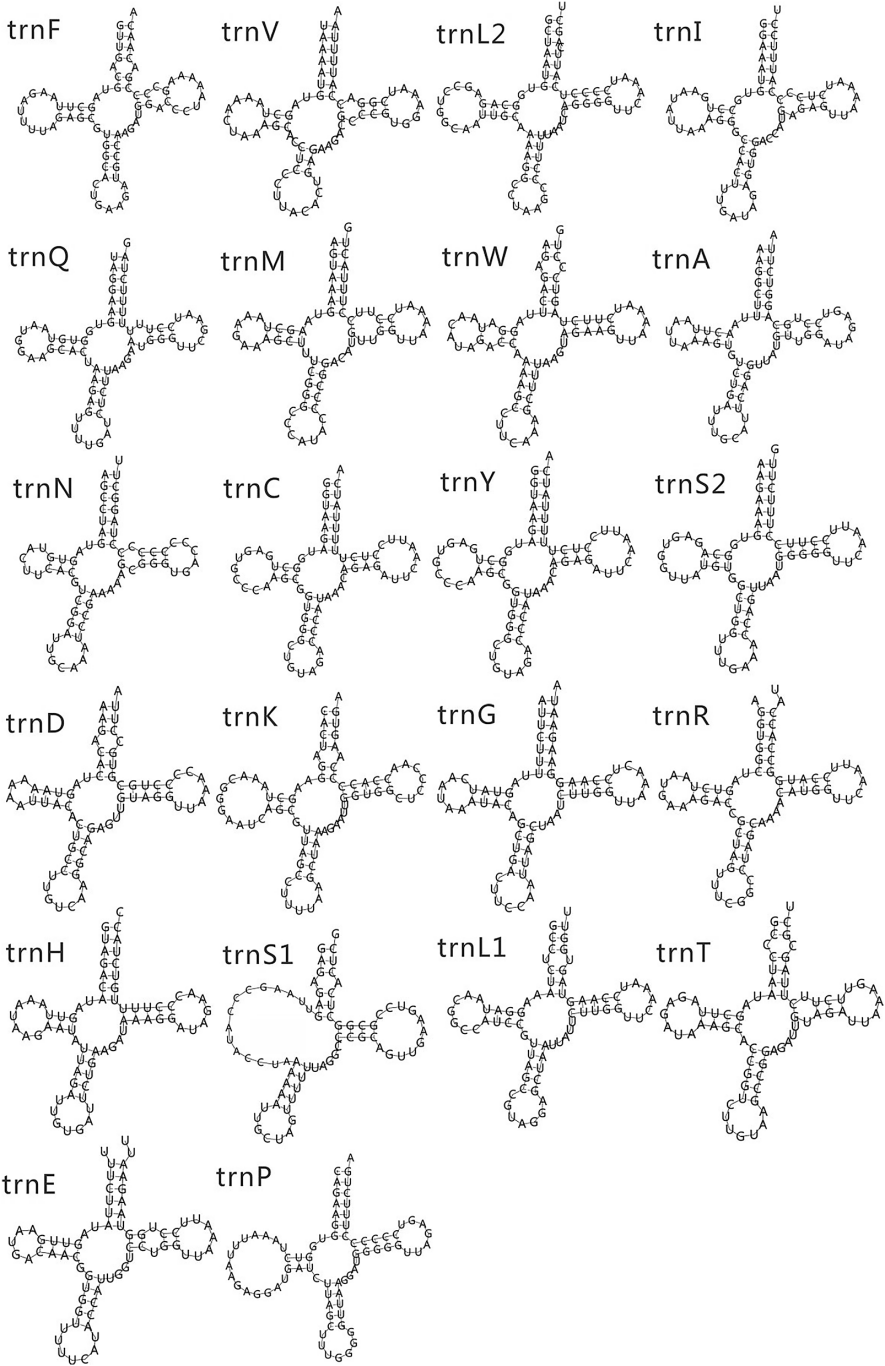
The secondary structure of tRNA in the mitochondrial genome of *Muraenesox cinereus*.

There were also 22 tRNAs in the mitochondrial genes of *M. cinereus*, like those of other vertebrates. Of these 22 tRNAs, 14 tRNAs were encoded on the heavy chain and the remaining eight (*tRNA-Gln, tRNA-Ala, tRNA-Asn, tRNA-Cys, tRNA-Tyr, tRNA-Ser, tRNA-Glu and tRNA-Pro*) were encoded on the light chain (Fig. 1). Among the 22 tRNAs, except that *tRNA-Ser*^*UCA*^ lacks the entire dihydrouridine arm, all other tRNAs have a typical clover structure (Fig. 3). In this case, *tRNA-Ser* lacks the dihydrouridine arm and is often seen in the mitochondrial of other vertebrates^13,52^. The length of *M. cinereus* mitochondrial tRNA is 1564 bp, and the length of 22 tRNA is between 66 and 76 bp; the base composition was A = 32.0%, T = 25.3%, C = 23.1% and G = 19.6% (Tables 1, 3). The AT and GC skew values of tRNA genes were 0.116 and − 0.081, respectively, which indicated that As and Cs were more abundant than Ts and Gs. The origin of light chain replication (OL) was usually located within a WANCY cluster, approximately two-thirds of the genomic distance from CR, and can fold into a stable stem ring secondary structure. Among the 22 tRNAs encoding 20 amino acids, two amino acids have two anti-codon sites, these two amino acids were Ser and Leu, and their anticodons were TGA/GCT and TAA/TAG, respectively. The small coding subunit (12S rRNA) and large coding subunit (16S rRNA) appeared on both sides with *trnF* and *trnL*^*(UUA)*^, which were located on the H-chain and separated by the *trnV* gene. The two rRNA genes are 2,668 bp in total length, and the base composition is A = 35.1%, T = 21.7%, C = 22.6%, and G = 20.6%. The AT-skew value was positive (0.236) and the GC-skew value was negative (− 0.047), which indicates that there were more adenine and cytosine nucleotides in rRNAs (Table 3).

**Figure 4:**
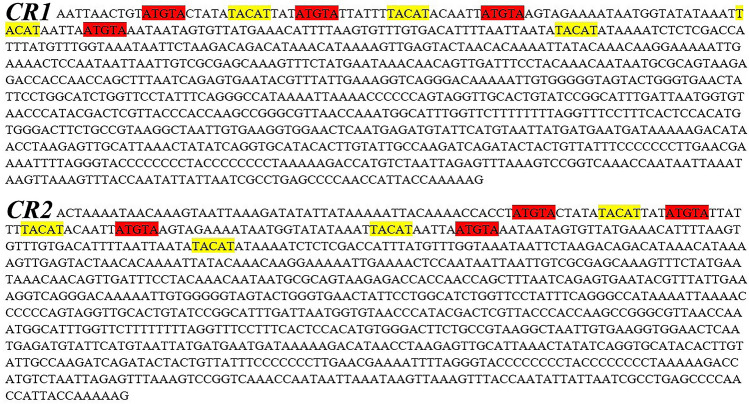
Compositional features of the control region of the *Muraenesox cinereus* mitochondrial genome. Palindromic motif sequence “TACAT’ and ‘ATGTA’ are marked in yellow and green respectively.

The lengths of the two CRs were 902 bp and 944 bp, respectively, and the total length was 1846 bp, of which the ratio of AT was 66.3% (Tables 1, 3). The AT ratio of the CR region is higher than that of other parts of mitochondrial genes, so the CR region is also called the "AT-rich region", which was also common in the mitochondria of other fish. Both AT and GC skew values were 0.092 and − 0.066, indicating that the number of adenine and cytosine nucleotides was higher than that of thymine and guanine nucleotides. The palindrome sequence motifs "TACAT" and "ATGTA" related to the termination of heavy chain replication were found in both CRs, and had been reported in other study^56^ (Fig. 4).

### Gene rearrangement

**Figure 5:**
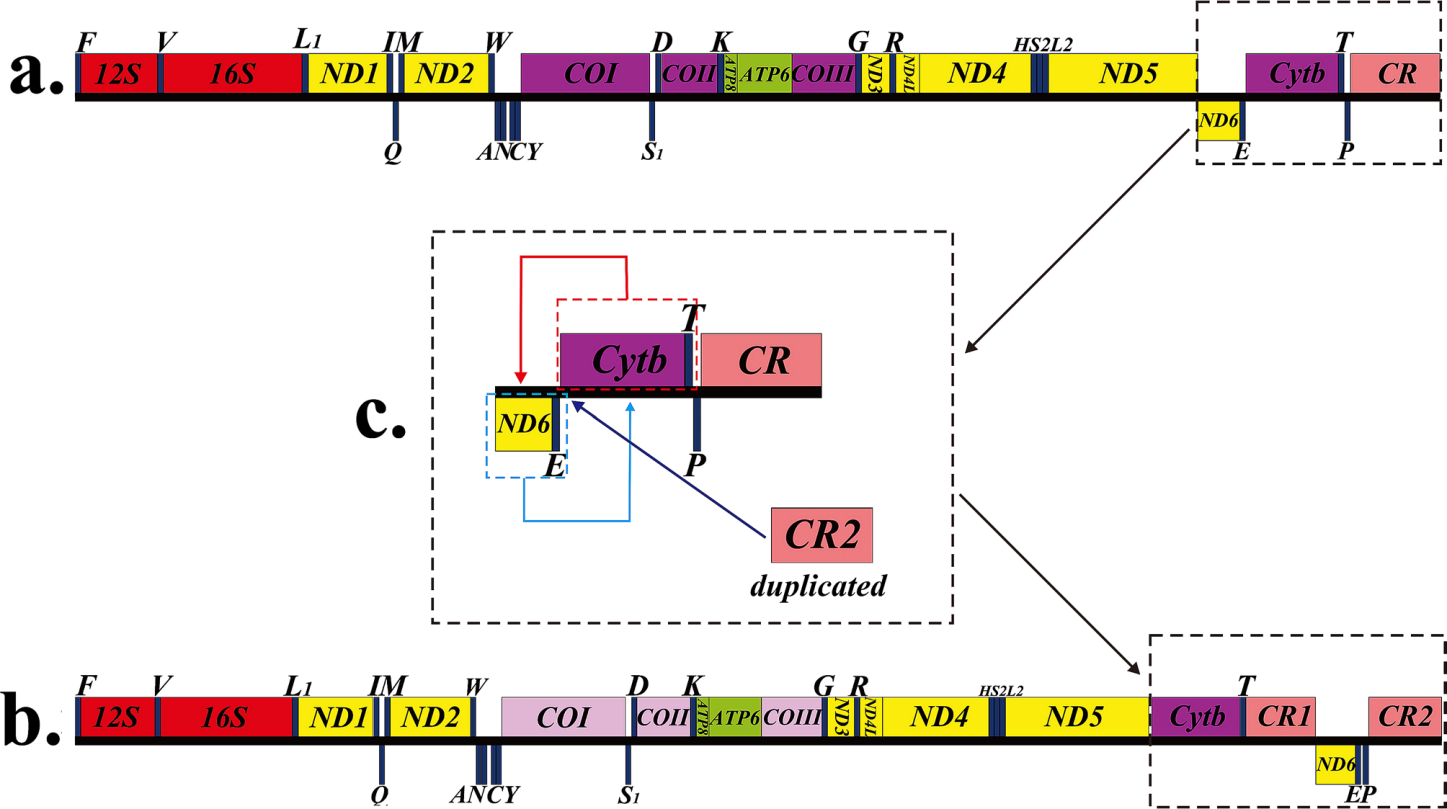
Analysis of *Muraenesox cinereus* mitochondrial gene rearrangement.

Compared with the gene arrangement in the vertebrate mitochondrial genome, the gene order in the moray mitochondrial genome obviously rearranged^57^ (Fig. 1). The position of the three genes (*ND6*, *trnE* and CR) in the moray *M. cinereus* mitochondria had changed. In general, *ND6* and the bound *trnE* were located between the *ND5* and *Cytb* genes, and only one CR region was located at the end of the mitochondrial genome. However, the position of *ND6* and *trnE* in the mitochondrial genome of *M. cinereus* changed, and a CR was copied (Fig. 5). In this study, except for *ND6*, *trnE* translocation and CR repeat, the remaining gene sequence was the same as the gene sequence in the vertebrate mitochondrial genome (Fig. 5).

How did the rearrangement occur in the *M. cinereus* mitochondrial genome? Considering the gene rearrangement model previously described in this study, we must consider which model is best suited to explain the gene rearrangement observed in the *M. cinereus* mitochondrial genome. Recombination model was only suitable for the exchange and reversal of small fragments, and this model was relatively rare in the mitochondrial genome^58,59^. Therefore, this recombination model is not suitable for explaining the mitochondrial gene rearrangement of *M. cinereus*. Regarding the TDNL and DRRL models, these two models were most used in genome rearrangement, where genes were clustered with the same polarity (L- or H-chain coding) and their relative order has not changed^27,43^. Therefore, the two models are also not suitable to explain the phenomenon of molecular rearrangement of *M. cinereus*. This tandem duplication and random loss (TDRL) model explains well the rearrangement of genes with redundant genes. The TDRL model was due to the incomplete deletion of repeated genes leading to the existence of intergenic spacers or pseudogenes^23,60,61^. Therefore, in this study, the TDRL model was suggested for rearrangement events, because gene rearrangements with repeated CRs were observed in the mitochondrial genome of *M. cinereus*, as described previously in the mitochondrial genome of parthenogenetic lizards^62^. The evidence of the TDRL model was indicated by the presence of pseudogenes or duplicate genes and the position of the gene spacer. There are four intervals in the rearrangement region of the mitochondrial genome of moray eel, located between *ND5* and *Cytb*, *trnT* and *CR1*, *ND6* and *trnE*, *trnE* and *trnP*, this phenomenon provides the basis for this model (Table 2).

Based on the principle of parsimony, we made assumptions about the region of the moray mitochondrial genome rearrangement, assuming the intermediate steps of gene rearrangement as follows. First, after the normal mitochondrial gene block (*ND6-trnE-Cyt b-trnT-trnP-*CR) is completely replicated, a gene block (*ND6-trnE-Cyt b-trnT-trnP-*CR*-ND6-trnE-Cyt b- trnT-trnP-*CR) (Fig. 5a,b). Then, after consecutive copies, other genes (*ND6*, *trnE*, *Cyt b*, *trnT* and *trnP*) were randomly lost in addition to CR. After the copied genes are randomly lost, the positions of the two genes in the red dotted box and the two genes in the cyan dotted box in Fig. 5c are swapped, and then inserted after the two genes in the red dotted box CR2. Therefore, after such copying and random loss, both CRs are preserved. We speculate that both CRs retain their original functions. In other animal mitochondrial genomes, similar hypotheses have been proposed^47^.

### Phylogenetic analysis

**Figure 6:**
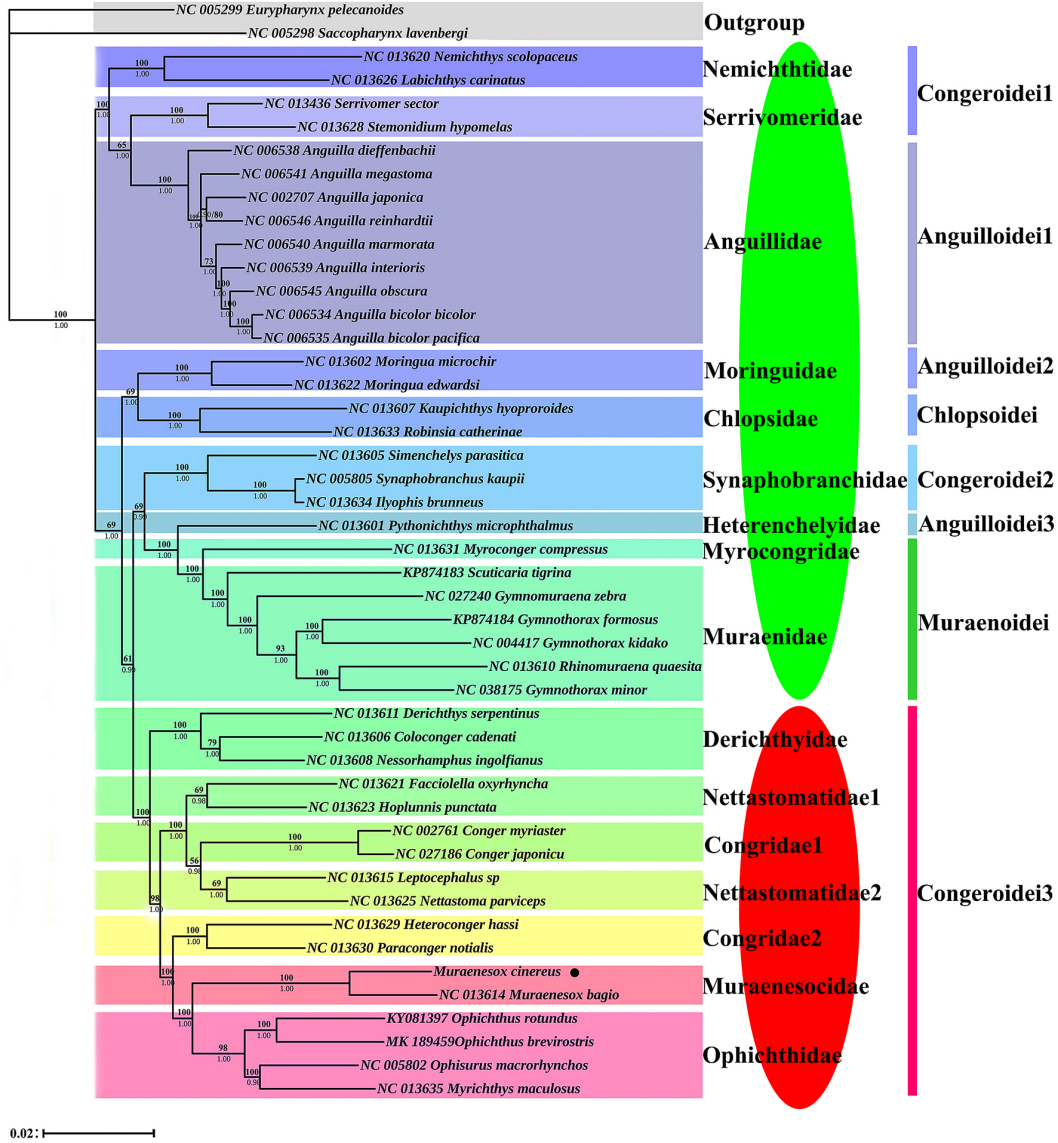
Phylogenetic analysis based on the nucleotide sequences of the 12 PCGs in the mitogenome. The numbers beside the nodes are posterior probabilities (BI, bottom) and bootstrap (ML, top). Red ellipse: gene rearrangement occurs; cyan ellipse: no gene rearrangement occurs.

To further study the evolutionary status of *M. cinereus* in the Anguillaridae, we selected 14 closely related families and two outgroups (*Saccopharynx lavenbergi* and *Eurypharynx pelecanoides*^63^) to construct evolutionary trees (BI and ML) to analyze phylogenetic relationships. After removing highly differentiated regions, a phylogenetic tree was constructed with 10,987 bp sequence. The results show that the topological structure of the ML tree and the BI tree are basically the same. Therefore, we merge two trees together to form a tree. In addition, the BI tree has a higher support value than the ML tree (Fig. 6). Both trees clearly show that *M. cinereus* and *M. bagio* were the closest in relationship, and that these two species form the Muranesocide branches (BI posterior probabilities [PP] = 1; ML bootstrap [BP] = 100). The mitochondrial genome structures of *M. cinereus* and *M. bagio* were very similar. However, there was no other gene between *tRNA-Thr* and *ND6* gene in *M. bagio*^64^ mitochondrial genome, but CR1 gene existed between *tRNA-Thr* and *ND6* gene in *M. cinereus* mitochondrial genome. In the *M. bagio* mitochondrial genome, *ND6* combined with *tRNA-Glu* rearranged and transferred between the *tRNA-Thr* and *tRNA-Pro* genes. However, in the mitochondria of *M. cinereus*, not only *ND6* and *tRNA-Glu* were rearranged, but also the CR gene was rearranged. Inoue et al.’s^64^ complete mitochondrial data studies and Santini et al.’s^7^ tandem dataset (mitochondrial and nuclear genes) studies also support Congridae’s non-singularity^9,65^. Regarding the unity of Nettastomatidae, the ML tree showed that all Nettastomatidae species were grouped into a clade, and supported the origin of single lines. However, the BI tree divided Nettastomatidae into two clades, indicating that Nettastomatidae was non-singleton, which was consistent with the results of Inoue^64^ and Lu et al^13^. Our results indicated that both Derichthyidae and Chlopsidae were monophyletic, but Santini et al.'s^7^ results were contrary to ours. For the main relationship between the Anguilliformes lineage and different families, our results were basically consistent with previous molecular studies^66,67^.

In our research, it was found that Chlopsoidei, Muraenoidei and Anguilloidei all had typical vertebrate mitochondrial genome sequences, but Congroidei had two different patterns of gene arrangement: (1) with typical vertebrate gene order (Nemichthyidae, Serrivomeridae and Synaphobranchidae) (2) with gene rearrangement (Derichthyidae, Nettastomatidae, Congridae, Muraenesocidae and Ophichthidae). The four families (Ophichthidae, Derichthyidae, Muraenesocidae and Nettastomatidae) studied by Inoue et al.^8^ also had mitochondrial genes lacking *ND6* and *trnE*, which were also clustered, which was also consistent with our results. In this study, we found an interesting phenomenon. The five families with gene rearrangement at the bottom formed a branch, and those without gene rearrangement formed a separate branch at the top of the tree (The cyan ellipse in Fig. 6 indicates that no gene rearrangement had occurred; the red ellipse indicated that gene rearrangement had occurred). These results indicate that the origin of eels is a diverse evolution. If the new gene sequence originates from a single ancestral species in the Congroidei suborder, then this pattern of presence/absence may be a good phylogenetic marker for identifying monoline populations as Kumazawa and Nishida^68^ and Macey et al^18^ suggested, they had a higher vertebrate relationship. Therefore, more advanced methods can be considered to classify the controversial Anguilliformes species. There are still some phylogenetic mismatches based on morphological and molecular data, so more eel mitochondrial genomes should be sequenced to support this hypothesis in future research.

## Conclusion

With the advancement of genetics research, many people believe that the richness of molecular information is superior to morphological data, and molecular analysis has become the most commonly used methods in the study of biological system development. Therefore, in this study, we sequenced and assembled the complete mitochondrial gene of *M. cinereus* and described its characteristics, which contains 37 genes and two control regions. After comparing with the typical vertebrate mitochondrial genes, we found that *M. cinereus* mitochondrial gene rearrangement obviously occurred, the rearrangement part of the gene *ND6* and *trnE* were transferred between *trnT* and *trnP*, accompanied by CR repeat. The most suitable model to explain this rearrangement phenomenon is the duplication-random loss model. The two phylogenetic trees (BI and ML) created using the mitochondrial genomes from 46 Anguilliformes were basically consistent with previous molecular studies on the interrelationships between the main Anguilliform lineages and different families, although some of these families were slightly related different. Both phylogenetic trees strongly support the non-monophyly of Congridae, providing a basis for the more advanced classification of Anguilliformes. In addition, our research results provide a theoretical basis for in-depth understanding of the mechanism and evolution of *M. cinereus* gene rearrangement and phylogenetic studies of eel.

## Materials and methods

### Sample collection permit and experimental approval

All procedures in this study were performed under the guidelines of the Regulations for the Administration of Laboratory Animals (Decree No. 2 of the State Science and Technology Commission of the People's Republic of China, November 14, 1988), and were approved by the Animal Ethics Committee of Zhejiang Ocean University (Zhoushan, China).

### Fish sample, DNA extraction, PCR amplification and sequencing

Individual *M. cinereus* specimens were collected by a commercial trawl fishing method in Zhoushan City, Zhejiang Province, China (30° 40′ 30″ N, 121° 20′ 28″ E) and immediately preserved with 95% ethanol. Total genomic DNA was extracted using the SQ tissue DNA kit (OMEGA) according to the manufacturer's protocol. After extraction, the DNA was stored in − 4 ℃ refrigerator. The polymerase chain reaction (PCR) primers used in this experiment designed 10 pairs of primers for the amplification of the complete mitochondrial genome of *M. cinereus* based on the complete mitochondria published by the predecessors^13,69,70^ (Table S1). The PCR was carried out in a 25 μl reaction volume containing 2.0 mM MgCl_2_, 0.4 mM of each dNTP, 0.5 μM of each primer, 1.0 U of Taq polymerase (Takara, China), 2.5 μl of 10 × Taq buffer, and approximately 50 ng of DNA template. Using the following cycling conditions: (1) initial activation step for 5 min at 95 °C; (2) 35 cycles of denaturation at 95 °C for 30 s, annealing at 52 °C (as the case may be) for 30 s and extension at 72 °C for 30 s; and (3) a final extension of 5 min at 72 °C. The sequences were determined using an ABI genetic analyzer (Applied Biosystems, China).

### Sequence assembly, annotation and analysis

The obtained sequence fragment was passed through CodonCode Aligner 9.0.1 (CodonCode Corporation, Dedham, MA) was assembled into a complete mitochondrial genome. The assembled mitochondrial genome is annotated by Sequin (version 15.10, http://www.ncbi.nlm.nih.gov/Sequin/). The boundaries of protein coding and ribosomal RNA genes were determined by NCBI-BLAST (http://blast.ncbi.nlm.nih.gov). The tRNA genes were verified using MITOS WebServer (http://mitos2.bioinf.uni-leipzig.de/index.py) using the default setting^49^. Composition skew values were calculated according to the following formulas^71^: AT skew = (A − T)/(A + T); GC skew = (G − C)/(G + C). The base composition and relative synonymous codon usage (RSCU) were obtained using MEGA X^72^. The mitochondrial gene map of *cinereus* was generated online by using CGView^73^.

### Phylogenetic analyses

Download 46 complete Anguilliformes mitochondrial genomes from GenBank (https://www.ncbi.nlm.nih.gov/genbank/) for phylogenetic studies (Table 1). Saccopharyngiformes was considered to be an intimately related species to Anguilliformes^13,74^; therefore, we selected two species of *Neocyema erythrosoma* and *Saccopharynx lavenbergi* in Saccopharyngiformes as the outgroup in this study. The 12PCGs sequences used for phylogenetic analysis were extracted from DAMBE version 7.2.3^75^. The 13 PCGs used did not include *ND6* and were not used because of their heterogeneous base composition and consistent poor phylogenetic performance^33,76^. Sequences were aligned with default parameters using Clustal X 2.0^77^, and manually checked using BioEdit^78^. Use software Gblock^79^ to eliminate ambiguous sequences. Substitution vs. the *Tamura-Nei* (TN93) genetic distance in pairwise comparisons was used to test for substitution saturation using DMABE version 7.2.3^75^. The third codon position shows significant saturation (Fig. S1), so it is only defined as purine and pyrimidine. Based on Bayesian inference (BI) and maximum likelihood (ML) two methods, using PhyML^80^ and MrBayes 3.2.6^81^ software for phylogenetic analyses. Based on the Akaike information criteria (AIC), parallel uses Modeltest 3.7^82^ of 56 models for ML analysis, and MrModeltest 2.2^83^ of 24 models for BI analysis to determine the best evolutionary model and points out that GTR + G + I was the analysis dataset of best-fit alternative models. Perform Bootstrap analysis (1,000 repetitions) to assess the relative level of support for ML analysis^84,85^. Use "Lset" and "Prset" for Bayesian phylogenetic analysis and allow the program to converge to the best estimate of model parameters. Other parameter settings are as follows: each Markov chain starts from a random tree, runs for 2 million generations, and samples a tree every 100 generations (a total of 20,000 trees are sampled) to ensure the independence of the samples. In order to improve the mixing ability of the Markov chain, the Metropolis coupling Markov chain Monte Carlo (MCMCMC) method was used, and three heating chains (temperature = 0.5) and one cold chain were operated simultaneously. To guarantee the stationarity had been reached, the average standard deviation of split frequencies was set below 0.01. The phylogenetic tree was viewed in FigTree v1.4.0.

### Ethical standards

Ethics Committee approval was obtained from the Institutional Ethics Committee of Zhejiang Ocean University to the commencement of the study.

## Supplementary Information

Supplementary Figure S1.

Supplementary Information.
