# Supplementary figures and images for: Novel gene rearrangement in the mitochondrial genome of Muraenesox cinereus and the phylogenetic relationship of Anguilliformes
Source: Sci Rep. 2021 Jan 28;11:2411. doi: 10.1038/s41598-021-81622-9 (PMC7844273; doi:10.1038/s41598-021-81622-9)

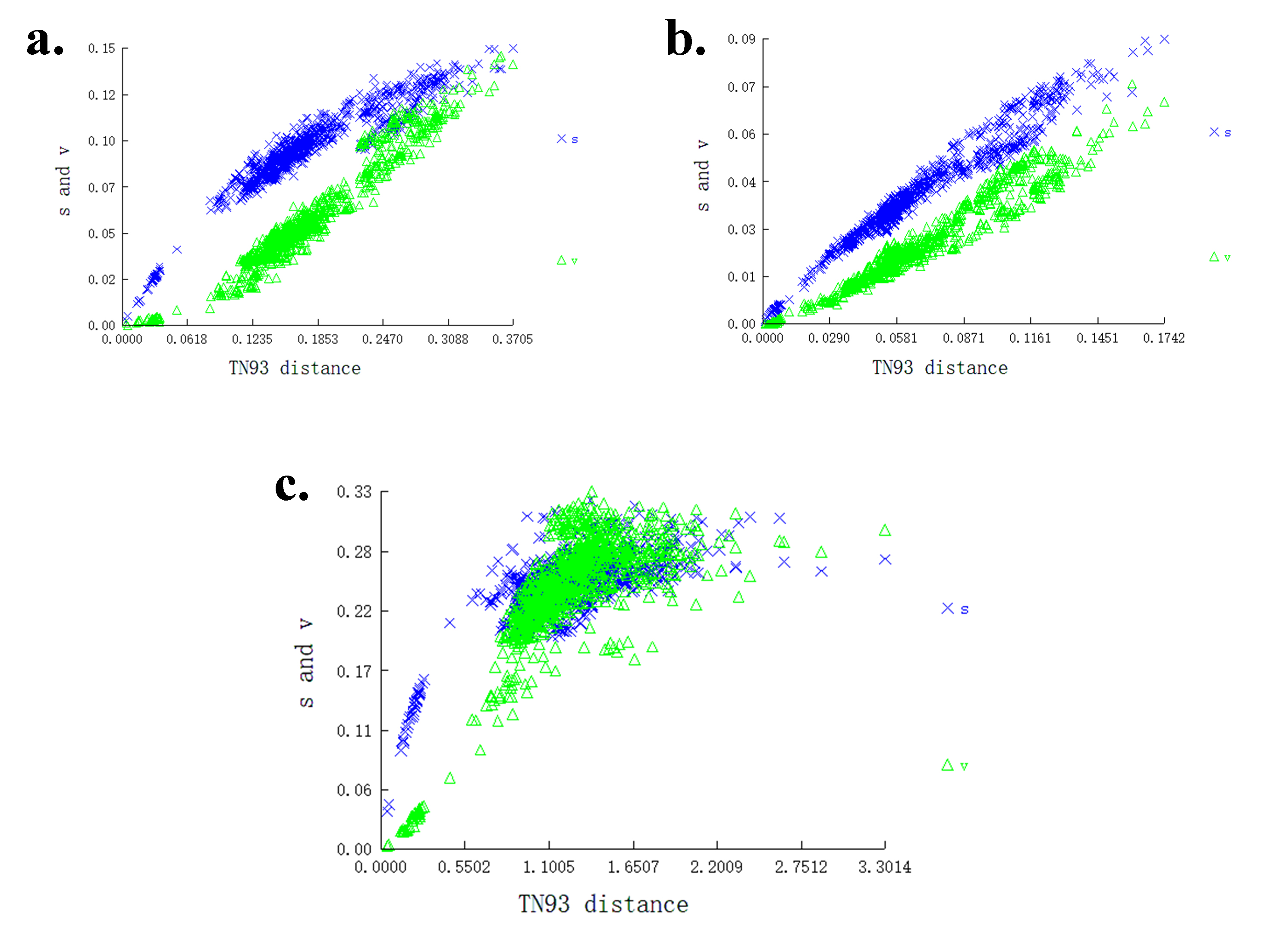

Supplement: Supplementary file 1 — Supplementary Figure S1. [file 41598_2021_81622_MOESM1_ESM.tif]
