# Supplementary material for: Novel gene rearrangement in the mitochondrial genome of Muraenesox cinereus and the phylogenetic relationship of Anguilliformes
Source: Sci Rep. 2021 Jan 28;11:2411. doi: 10.1038/s41598-021-81622-9 (PMC7844273; doi:10.1038/s41598-021-81622-9)
Supplement: Supplementary file 2 — Supplementary Information. [file 41598_2021_81622_MOESM2_ESM.docx]

**Novel gene rearrangement in the mitochondrial genome of** ***Muraenesox cinereus* and the phylogenetic relationship of Anguilliformes**

Kun Zhang^1,3^, Kehua Zhu^1,3^,Yifan Liu^1,3^*, Hua Zhang^2^, Li Gong^1,3^, Lihua Jiang^1,3^, Liqin Liu^1,3^, Zhenming Lü^1,3^, Bingjian Liu^1,2,3^*

1. National engineering Laboratory of Marine Germplasm Resources Exploration and Utilization, Zhejiang Ocean University, 316022, Zhoushan, China;

2. Key Laboratory of Tropical Marine Bio-resources and Ecology, Chinese Academy of Sciences

3. National engineering research center for facilitated marine aquaculture, Marine science and technology college, Zhejiang Ocean University, 316022, Zhoushan, China.

**Corresponding author(*)**

Yifan Liu, Bingjian Liu

Email: et999927@163.com (Y. Liu), [bjetbj@163.com](mailto:bjetbj@163.com)(B. Liu)

Address: No.1, Haida South Road, Zhoushan, Zhejiang, 316022 P.R.China

Tel: +86 580 8180386 Fax +86 580 8180386

| 引物名称 | 引物名称序列 |
| --- | --- |
| F1 | TGGCATCTGGCACGCATCAGTG |
| R1 | GACTAGGTGTGGAGACAGTCAGAC |
| F2 | ATCGCCTCTCGCTAACAAGCC |
| R2 | AGTGGAAGGATGACGAGTATGTT |
| F3 | CATTACACCACTTCCTAGTACAG |
| R3 | CTGATCACACGAATAGACGAG |
| F4 | CAACTTAGCTCATGCTGG |
| R4 | CTTCAATACCATTGATGGCC |
| F5 | CGCCCTTCCATCACTCCG |
| R5 | GAATTATGATCCTCATCAGTAA |
| F6 | GCTGCTGCATGATACTGACA |
| R6 | AATAAATCATCAAGCGGCTAT |
| F7 | CCCACGGACTAGTCTCATCC |
| R7 | CTTGTAGGGCTGCAGTATTAGCGT |
| F8 | ATTCCAACTATTCATCGGCTG |
| R8 | ATAGTGTATTGCTAAGAATAG |
| F9 | GACTCACTAGTTGACCTACC |
| R9 | GTAACGAGTCGTATGGGTTACA |
| F10 | GCATCTGGTTCCTATTTCAG |
| R10 | ATTGGTCGGTTATTTGACGAATT |
| F11 | CAAGATCAGATACTACTGTTATT |
| R11 | ACCAGACATATATGGTCGAGAGAT |
| F12 | AAGCTAAGATCATCCTCTTAAAT |
| R12 | TTATCTAATCACGCTTTACG |

Table S1 Primer design for mitochondrial genome amplification of *Muraenesox cinereus*


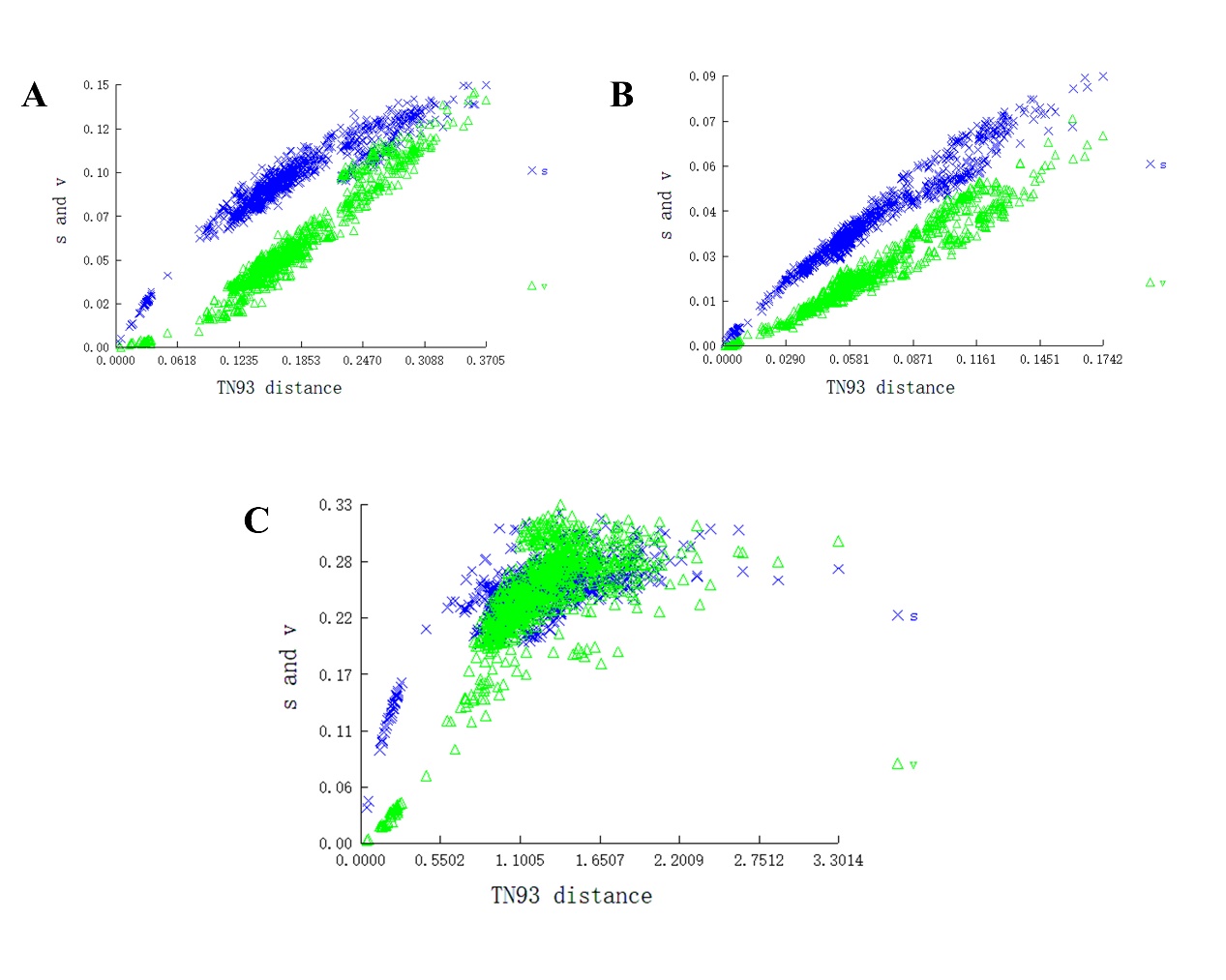


Fig. S1. Patterns of sequence variations of mitogenomic data comprising concatenated nucleotide sequences from the 12 protein-coding genes (excluding the ND6 gene) from 47 species. Transitional (TS) and transversional (TV) differences are plotted against the Tamura-Nei (TN93) genetic distance. A) The first codon position; B) the second codon position; C) the third codon position.
